# Regulation of *COL1A2*, *AKT3* genes, and related signaling pathway in the pathology of congenital talipes equinovarus

**DOI:** 10.3389/fped.2022.890109

**Published:** 2022-07-22

**Authors:** Ningqing Wang, Jiangchao Zhang, Haixiang Lv, Zhenjiang Liu

**Affiliations:** Department of Orthopedics, Children’s Hospital, Capital Institute of Pediatrics, Beijing, China

**Keywords:** congenital talipes equinovarus, *COL1A2*, *AKT3*, differentially expressed genes, key genes

## Abstract

Congenital talipes equinovarus (CTEV) is one of the most common congenital limb defects in children, which is a multifactorial and complex disease that associates with many unknown genetic, social-demographic, and environmental risk factors. Emerging evidence proved that gene expression or mutation might play an important role in the occurrence and development of CTEV. However, the underlying reasons and involved mechanisms are still not clear. Herein, to probe the potential genes and related signaling pathways involved in CTEV, we first identified the differentially expressed genes (DEGs) by mRNA sequencing in pediatric patients with CTEV compared with normal children. The gene of *COL1A2* was upregulated, and *AKT3* was downregulated at the transcriptional level. Western blot and quantitative polymerase chain reaction (qRT-PCR) results also showed that the expression of *COL1A2* in CTEV was enhanced, and the *AKT3* was decreased. Furthermore, the *COL1A2* Knock-in (+*COL1A2*) and *AKT3* Knock-out (-*AKT3*) transgenic mice were used to verify the effects of these two genes in the CTEV, and the results of which showed that both *COL1A2* and *AKT3* were closely related to the CTEV. We also investigated the effect of the PI3K-AKT3 signaling pathway in CTEV by measuring the relative expression of several key genes using Western blot and qRT-PCR. In line with the Kyoto Encyclopedia of Genes and Genomes (KEGG) analysis data, the PI3K-AKT3 signaling pathway might play a potentially important role in the regulation of pathological changes of CTEV. This study will provide new ideas for the mechanism investigation and prenatal diagnosis of CTEV.

## Introduction

Congenital talipes equinovarus (CTEV), also known as clubfoot, is one of the most common congenital limb defects in children that is characterized by hindfoot varus, forefoot adduction, high arch foot, and plantar flexion deformity (1). The epidemiological data suggest that the incidence of CTEV is nearly 0.5–2‰, and the number of male patients is twofold that of the female patients (2, 3). In low- and middle-income countries, the prevalence of CTEV seems higher than the developed countries, and the incidence of CTEV is racial differences (1, 4). Based on the clinical presentation, CTEV can be classified as secondary, syndromic, and idiopathic. It has been reported that 20% of the cases can be secondary or syndromic when associated with other congenital diseases, and 80% of cases may happen as birth defects without any relationships with other malformations (1). The traditional treatment for CTEV is early postnatal non-surgical therapy, such as the minimally invasive Ponseti method, which has been accepted as the initial treatment of CTEV in many countries (5). The Ponseti method has good mobility and functional outcomes, but the recurrence rate is up to ∼54% and may be accompanied by foot pain and muscle dysplasia in the surgery side leg (5, 6). Therefore, exploring the pathogenic causes and novel therapeutic methods has become a hot topic in CTEV research.

Characterization of the protein composition in the extracellular matrix of contracted tissue is important and helpful in understanding the pathogenesis of CTEV. In total, 13 infant patients with idiopathic clubfoot were selected to analyze the protein composition of the extracellular matrix in contracted tissue by proteomic analysis. The extracellular matrix in clubfoot was composed of additional 16 proteins including collagens V, VI, and XII, as well as collagens I, III, and transforming growth factor β (7). Kerling et al. also examined whether there were quantitative changes of key proteins in the extracellular matrix by immunohistological analysis, resulting in that the values of collagens I, III, VI, and undulin in musculus gastrocnemius were higher than musculus vastus lateralis, while the value for tissue inhibitor of matrix metalloproteinase-2 was reduced (8). There were no significant differences in the components of the musculus tibialis anterior and musculus vastus lateralis, as well as no differences between the male and female patients or between the patients who suffered on one side and both sides (8). Moreover, emerging studies indicated that CTEV is a multifactorial and complex disease that is not only caused by the interaction of many unknown genetic factors but also social-demographic and environmental risk factors (1, 2, 9). Theories about genetic factors include abnormal extracellular matrix, neuromuscular injury, skeletal dysplasia, soft tissue contracture, vascular abnormalities, Bacino et al. (2). Several abnormally expressed genes were proved closely associated with the CTEV, such as homeobox gene (*HOX*), collagen family genes (*COL*), cysteinyl aspartate specific proteinase (CASP), and so on (7, 10). For example, *HOX* cluster has been proved to play an important role in regulating muscle development of children and also can regulate the simultaneous development of muscles, tendons, and cartilage (11, 12). In one clinical study, the mRNA of *COL9A1* and *COL1A1* in patients with CTEV was remarkably higher than normal people, which suggested that the *COL* family might associate with the CTEV (13). However, the underlying reasons and involved mechanism of CTEV are still not completely clear.

In recent years, gene sequencing technologies including whole-genome linkage analysis, mRNA sequencing, gene chip, and whole-exome sequencing have provided bases for the investigation of gene pathways, developmental processes, and environmental factors on the pathogenesis of CTEV. Especially, RNA sequencing technology has been widely used in the research of various human diseases, and it is the most widely used research mode of transcriptomics in disease research on the changes of genes at the transcriptome level (14). Moreover, RNA sequencing can rapidly and massively supplement the biological information of patients with CTEV, which is helpful to carry out in-depth molecular biology research on CTEV. In this study, we probed the differentially expressed genes (DEGs) in child patients with CTEV compared with normal children by mRNA sequencing. Then, the target gene Knock-in (KI) or Knock-out (KO) mice were constructed to identify the genes’ function and related signaling pathways in the CTEV. This study provides new ideas for the mechanism investigation and prenatal diagnosis of CTEV.

## Materials and methods

### Materials

The plasma samples of pediatric patients with CTEV and normal children were collected from the Department of Pediatric Orthopedics, Shengjing Hospital, China Medical University. Both the groups included 1 man and 2 women, and the ethnicity of all children was Han Chinese. The children in the control group had no history of CTEV and other congenital skeletal and musculoskeletal malformations and without any abnormal blood biochemical indexes. The CTEV and normal children aged in the range from 4 to 6 years. All patients in this study met the inclusion and exclusion criteria, and all clinical specimens were collected with the informed consent of the children’s guardians and approved by the Medical Ethics Committee of Shengjing Hospital affiliated with China Medical University (No. 2017PS226K). All the reagents were purchased from Beijing Chemical Plant (Beijing, China) without any special notes.

### Experimental animals

Both the wild-type (WT) C57BL/6 mice and transgenic mice were generated by Cyagen Biosciences (Guangzhou, China). The mice were hosted following guidelines of the Institute for Experimental Animals of Shengjing Hospital affiliated with China Medical University and were approved by the Medical Ethics Committee for animal experiments. The relative humidity of the mouse room is 55 ± 5%, with a temperature of 25°C and a 12 h/12 h light/dark cycle. The fetal mice were obtained and checked about the CTEV-like morphology after the female mice conception for 21 days.

### mRNA sequencing

Three pediatric patients with CTEV and control children were recruited for the identification of key genes involved in CTEV, in which, the male to female ratio was 1:2. A volume of 10 ml peripheral venous blood was collected from 7 to 9 a.m. Then, the blood was centrifuged at 1,000*g* for 10 min. After removing the pellet, plasma was obtained and stored at −80°C. The plasma samples were sent to Aksomics Biotechnology Co., Ltd., (Shanghai, China) to perform the mRNA sequencing. In brief, the total RNA of the plasma sample was isolated using TRIzol reagents (Promega, United States) and identified using agarose gel electrophoresis and microspectrophotometer. After the RNA library was constructed by 1∼2 μg RNA and identified by Agilent 2100 Bioanalyzer, the sequencing was carried out using Illumina NextSeq 500 sequenator. Gene ontology (GO) and Kyoto Encyclopedia of Genes and Genomes (KEGG) pathway were used to analyze the potential gene functions and signaling pathways.

### Western blot

The plasma was isolated by centrifuging at 1,000*g* for 10 min. Then, the total protein concentration was detected using the bicinchoninic acid (BCA) method. Notably, 5 μg/μl plasma samples were boiled for 5 min after mixing with 2xSDS-PAGE loading buffer and then loaded on SDS-PAGE prefabricated gel (8%) for gel electrophoresis. The gel was transferred onto a nitrocellulose film at 100 V for 1.5 h. A total of 5% skimmed milk was used to block the membrane at room temperature for 1 h. The membrane was stained using the first antibody at 4°C on a shaker overnight. After rinsing three times with TBST buffer (pH 7.4), the second antibody was added and incubated at room temperature for 1 h. Finally, enhanced chemiluminescence (ECL) solution was added to the film, and the film was imaged using the TANON chemiluminescence instrument. Antibodies of COL1A2, AKT3, PI3K, mTOR, GSK3β, and MMP proteins were purchased from Abcam (Cambridge, MA, United States).

### Real-time quantitative polymerase chain reaction assay

The quantitative polymerase chain reaction (qRT-PCR) was performed following standard procedures (15). The total RNA of the plasma sample was isolated using TRIzol reagents following the manufacturer’s instructions. Then, the first-strand cDNA was prepared using the Hi Fi-Script First Strand cDNA Synthesis Kit (Kangwei, Beijing, China). Then, the RT-PCR was carried out using a real-time PCR system (Bio-Rad, Hercules, CA, United States) following the amplification parameters: pre-denaturation at 95°C for 10 min, 40 cycles of denaturation at 95°C for 15 s, and annealing at 60°C for 30 s. β-actin was used as an internal control. The relative expression of genes between the CTEV and control samples was calculated using the following equation: fold changes = 2^–ΔΔ*Ct*^ (16). All the primers for qRT-PCR are listed in Table 1.

**TABLE 1:** Primer sequences used in the quantitative polymerase chain reaction (qRT-PCR) assay.

Gene	Direction	Primer sequence (5′-3′)
*AKT3*	Forward	ATAATCAGATGTCTCCAGTGGAC
	Reverse	ATAATCAGATGTCTCCAGTGGAC
*COL1A2*	Forward	GAAGGCTCTAGAAAGAACCC
	Reverse	CCAGTAGTAACCACTGCTC
*GSK3*β	Forward	GACTAAGGTCTTCCGACCCC
	Reverse	TTAGCATCTGACGCTGCTGT
*MMP*	Forward	AAGGGTACAGCCTGTTCCTGGT
	Reverse	CTGGATGCCGTCTATGTCGTCT
*mTOR*	Forward	TTCATTCTTTCATTGGAGACGG
	Reverse	CTCGAACCCTGTTAATAATCTGG
*PI3K*	Forward	ACTGGCAACCCAGAACTGATA
	Reverse	CCGAGACACCACAGCTGAAT
β*-actin*	Forward	ATATCGCTGCGCTGGTCGT
	Reverse	CCTTCTGACCCATTCCCACC

### Statistical analysis

The data were presented as (means ± SD). The difference analysis of different groups was performed by one-way ANOVA followed by Tukey’s test; *p < 0.05; **p < 0.01; ***p < 0.001; ****p < 0.0001.

## Results

### Identification of the key genes involved in congenital talipes equinovarus by mRNA sequencing

**FIGURE 1:**
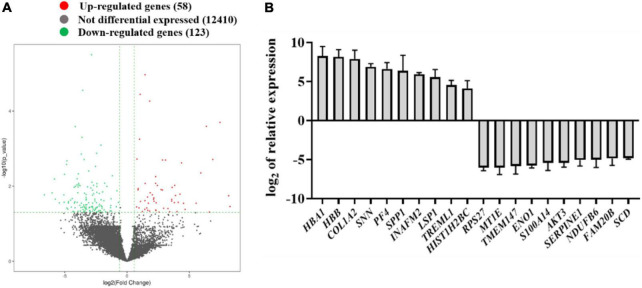
Identification of the differentially expressed genes in the plasma from normal children and child patients with congenital talipes equinovarus (CTEV) using mRNA-seq. **(A)** Volcano plot of gene expression changes between control and child patients with CTEV. **(B)** Top 10 upregulated and downregulated genes from the RNA-seq results.

The total RNA from CTEV and normal children’s plasma were extracted using TRIzol reagents (Promega, United States). After determining the purity and integrity of RNA samples by agarose gel electrophoresis and microspectrophotometer, the sequencing was carried out with the support of Aksomics Biotechnology Co., Ltd. (Shanghai, China). As shown in Figure 1A, scatter plots were used to evaluate the expression variations of mRNA between the CTEV and control samples. Totally, compared with the control group, 181 DEGs were screened in the CTEV group, in which 58 DEGs were upregulated, and 123 DEGs were downregulated. We calculated the relative expression of DGEs and collected the top 10 significantly expressed upregulated or downregulated genes (Figure 1B). Concretely, the top 10 most significantly upregulated genes were *HBA1*, *HBB*, *COL1A2*, *SNN*, *PF4*, *SPP1*, *INAFM2*, *LSP1*, *TREML1*, and *HIST1H2BC*, and the top 10 most significantly downregulated genes were *RPS27*, *MT1E*, *TMEM147*, *ENO1*, *S100A14*, *AKT3*, *SERPINE1*, *NDUFB6*, *FAM20B*, and *SCD*.

Gene ontology and KEGG pathway analysis were performed to predict the functional enrichment of differentially expressed mRNAs. In the GO analysis, three items including biological process, cellular component, and molecular function were conducted. The top 10 upregulated and downregulated GO terms are listed in Table 2, and we found that the upregulated process or function was mainly involved in hydrogen peroxide metabolic, oxygen transport, growth hormone, and extracellular process, and downregulated process mainly included cellular metabolic, RNA splicing, and nucleobase containing compound metabolic process. KEGG pathway analysis showed that malaria, cancer, and several other disease-related pathways corresponded to upregulated transcripts, and HIF-1 signaling, spliceosome, and PI3K-AKT signaling pathways corresponded to downregulated transcripts (Table 3).

**TABLE 2:** Gene ontology (GO) analysis of differentially expressed genes (DEGs) in the plasma from child patients with congenital talipes equinovarus (CTEV) compared with normal children.

	Biological process	Cellular component	Molecular function
Up-regulated	Hydrogen peroxide metabolic process	Extracellular region	Growth hormone activity
	Oxygen transport	Extracellular space	Growth hormone receptor binding
	Growth hormone receptor signal	Haptoglobin hemoglobin complex	Haptoglobin binding
	Cellular response to growth	Hemoglobin complex	Oxygen carrier activity
	Platelet degranulation	Platelet alpha granule	Receptor ligand activity
	Regulation of signaling recognition	Vesicle	Growth factor activity
	Extracellular structure organization	Cytoplasmic vesicle lemen	Extracellular matrix structure
	Hydrogen peroxide catabolic process	Platelet alpha granule lumen	Oxygen binding
	Regulated exocytosis	Secretory granule	Hormone activity
	Response to growth hormone	Secretory vesicle	Peroxidase activity
Down-regulated	Cellular metabolic process	Membrane bounded organelle	Peptide binding
	mRNA splicing *via* spliceosome	Nucleus	Amide binding
	RNA splicing *via* transeste	Intracellular membrane bounded	RNA polymerase I core binding
	Cellular nitrogen compound	Nucleoplasm	Protein binding
	Metabolic process	Intracellular organelle	Enzyme binding
	Nitrogen compound metabolic process	Intracellular	Organic cyclic compound binding
	Nucleobase containing compound metabolic	Nuclear lumen	RNA binding
	Cellular aromatic compound metabolic	Catalytic complex	SMAD binding
	Primary metabolic process	Catalytic step 2 spliceosome	RNA polymerase II activity
	RNA splicing	Cytoplasm	Disulfide oxidoreductase

**TABLE 3:** Kyoto Encyclopedia of Genes and Genomes (KEGG) pathway analysis of signal pathways of DEGs in the plasma from child patients with CTEV compared with normal children.

Up-regulated	Down-regulated
African trypanosomiasis	Bacterial invasion of epithelial
Cell adhesion molecules	Central carbon metabolism
ECM receptor interaction	Cytosolic DNA-sensing pathway
Focal adhesion	Epstein-Barr virus infection
Leishmaniasis	Glycolysis/Gluconeogenesis
Malaria	HIF-1 signaling pathway
Phagosome	Metabolic pathways
Proteoglycans in cancer	Mineral absorption
Pyrimidine metabolism	PI3K-AKT signaling pathway
Systemic lupus erythematosus	Spliceosome

Therefore, by combining the results of DEG candidates and predicting biological processes and pathways, we noticed that among the upregulated genes, the relative expression of *COL1A2* in CTEV was nearly 239-fold compared with the control group (Figure 1B). The *AKT3* gene was downregulated nearly 43-fold compared with the control group (Figure 1B), which is in line with the KEGG analysis results that the PI3K-AKT3 signaling pathway in the CTEV group was downregulated. All in all, we hypothesized that the gene of *COL1A2* was upregulated and *AKT3* was downregulated, both of which play a pivotal role in CTEV.

### *COL1A2* and *AKT3* were abnormally expressed in patients with congenital talipes equinovarus

**FIGURE 2:**
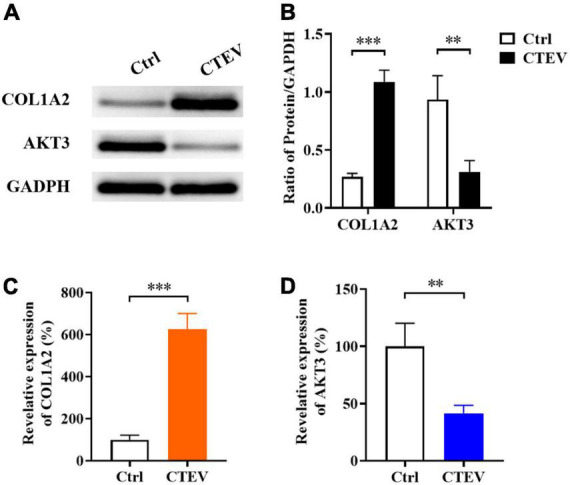
The expression of *COL1A2* and *AKT3* in the plasma from normal children and child patients with CTEV. **(A)** Western blot of plasma COL1A2 and AKT3 proteins. **(B)** Quantification of protein expression in panel **(A)**. Quantitative polymerase chain reaction (qRT-PCR) analyzation of plasma *COL1A2* **(C)** and *AKT3* **(D)** expression at the transcriptional level. Data are means ± SD (*n* = 3). Statistical significance was determined using one-way ANOVA followed by Tukey’s test; **p < 0.01; ***p < 0.001.

According to the mRNA sequencing and functional prediction, the DEGs of *COL1A2* and *AKT3* may play an important role in the CTEV. So, we detected the expressed proteins of COL1A2 and AKT3 in the plasma from normal children and child patients with CTEV using Western blot. As shown in Figure 2A, the dose of COL1A2 in the CTEV plasma group was obviously higher than the control group, and the AKT3 was lower than the control group, respectively. Quantification of the COL1A2 or AKT3 to GAPDH in the Western blot further proved that COL1A2 (*p* < 0.001) and AKT3 (*p* < 0.01) were significantly differentially expressed between the CTEV and control groups (Figure 2B).

We further probed the relative expression of *COL1A2* and *AKT3* at the transcriptional level using the qRT-PCR method. In accord with the Western blot results, the relative expression of *COL1A2* was significantly higher than the control group (*p* < 0.001), and *AKT3* was significantly lower (*p* < 0.01) (Figures 2C,D). Therefore, consistent with the mRNA sequencing results, it was indicated that the expression of *COL1A2* and *AKT3* in patients with CTEV were abnormal, in which, the *COL1A2* was higher expressed and *AKT3* was lower expressed compared with the normal people.

### Functional identification of *COL1A2* and *AKT3* in transgenic mice

**FIGURE 3:**
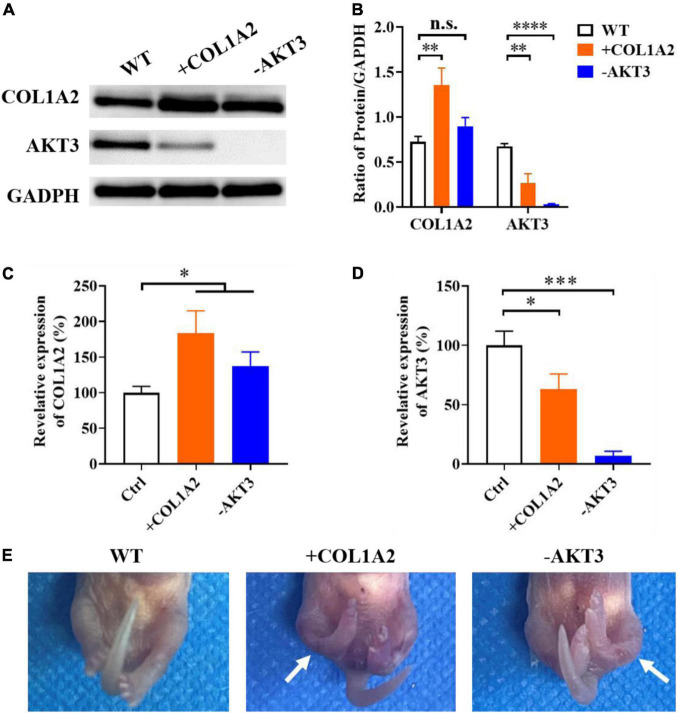
Functional identification of the target genes using *COL1A2* Knock-In (+ *COL1A2*) and *AKT3* Knock-Out (-*AKT3*) transgenic mice. **(A)** Western blot of plasma COL1A2 and AKT3 proteins. **(B)** Quantification of protein in panel **(A)**. qRT-PCR analyzation of plasma *COL1A2* **(C)** and *AKT3* **(D)** expression at the transcriptional level. **(E)** Morphological observation of the mice pups. Data are means ± SD (*n* = 3). Statistical significance was determined using one-way ANOVA followed by Tukey’s test; n.s., no significance; *p < 0.05; **p < 0.01; ***p < 0.001; ****p < 0.0001.

From the mRNA sequencing data and the expression measurement, we found that the genes of *COL1A2* and *AKT3* may play important roles in the occurrence and development of CTEV, and both of these two genes were abnormally expressed in the patients with CTEV. Therefore, we further used the *COL1A2* KI (+*COL1A2*) and *AKT3* KO (-*AKT3*) transgenic mice to determine the function of *COL1A2* and *AKT3* on CTEV. First, we obtained the +*COL1A2* and -*AKT3* transgenic mice according to the DNA prokaryotic microinjection method (17). After identifying the positive mutations by genotyping, we detected the expression of *COL1A2* and *AKT3* genes in the transgenic mice. As shown in the Western blot results, compared with the WT mice, the content of COL1A2 protein in the plasma was obviously higher, and AKT3 was lower in the +*COL1A2* transgenic mice (Figure 3A). It means that there may exist a relationship among the expression of *COL1A2* and *AKT3* genes, in which, the overexpression of the *COL1A2* gene may suppress the activation of *AKT3* expression. In the -*AKT3* transgenic mice, the content of COL1A2 protein in the plasma seemed less high than in the WT mice, while the AKT3 was scarcely detected (Figure 3A). Quantification of the COL1A2 or AKT3 blot to GAPDH proved that in the +*COL1A2* mice, the content of COL1A2 was significantly improved (*p* < 0.01), and AKT3 was reduced compared with the WT mice (*p* < 0.01) (Figure 3B). The content of COL1A2 was not significantly different in the -*AKT3* transgenic mice compared with the WT mice, while AKT3 was significantly decreased (*p* < 0.0001) (Figure 3B).

We also measured the relative expression of *COL1A2* and *AKT3* genes in both the +*COL1A2* and -*AKT3* transgenic mice using qRT-PCR. As shown in Figure 3C, the expression of *COL1A2* in both of these two transgenic mice was significantly increased than in WT mice at the transcriptional level (*p* < 0.05). However, the *AKT3* gene was significantly downregulated in both + *COL1A2* (*p* < 0.05) and -*AKT3* (*p* < 0.001) transgenic mice compared with WT mice (Figure 3D). As shown in Figure 3E, compared with the control fetal mouse, both the +*COL1A2* and -*AKT3* transgenic fetal mice had bilateral congenital clubfoot-like abnormalities. All the two lower limbs in the transgenic fetal mice were involved, and other morphological features of the CTEV phenotype including asymmetric hind limb, pelvic abnormalities, calcaneus varus, overlap of the talus, and calcaneus were also noted (Figure 3E) (3, 18). The CTEV phenotypes of +*COL1A2* transgenic fetal mice seemed more serious compared with the -*AKT3* mice (Figure 3E).

### The effects of PI3K/AKT3 signal pathway in the congenital talipes equinovarus

**FIGURE 4:**
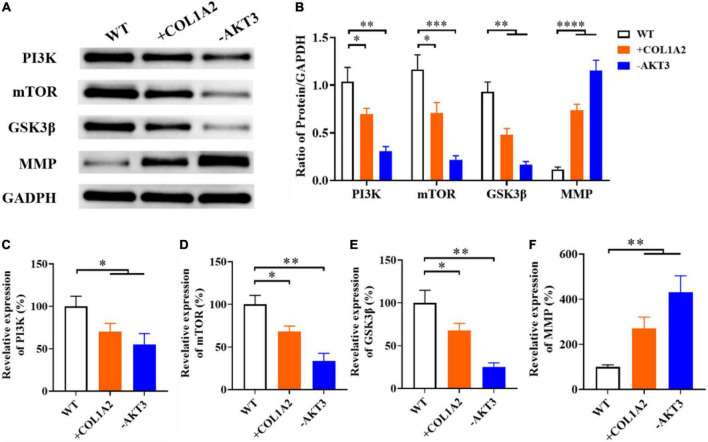
The effects of the PI3K/AKT3 signal pathway in the CTEV. **(A)** Western blot of the expression of key proteins involved in the PI3K/AKT3 signal pathway. **(B)** Quantification of protein in panel **(A)**. qRT-PCR analyzation of plasma genes of *PI3K* **(C)**, *mTOR* **(D)**, *GSK3*β **(E)**, and *MMP* **(F)** expression at the transcriptional level. Data are means ± SD (*n* = 3). Statistical significance was determined using one-way ANOVA followed by Tukey’s test; *p < 0.05; **p < 0.01; ***p < 0.001; ****p < 0.0001.

It was encouraging that *COL1A2* and *AKT3* genes were important in the pathological process of CTEV according to the above mRNA sequencing data of patients with CTEV and experiments on transgenic mice. We also wanted to know the effect of the related signaling pathway involved in the CTEV. According to the previous study, we detected the expression of key proteins or factors in the PI3K-AKT3 signaling pathway, including PI3K, mTOR, GSK3β, and MMP. As shown in Figure 4A, after KO of the *AKT3* gene, the content of all the three important proteins involved in the PI3K-AKT3 signaling pathway (PI3K, mTOR, and GSK3β) was obviously lower than the WT mice. While the MMP was remarkably abundant in the -*AKT3* transgenic mice (Figure 4A), similar results were occurred in the +*COL1A2* transgenic mice compared with WT mice (Figure 4A). Quantification of the PI3K, mTOR, GSK3β, and MMP blot to GAPDH showed that in both the +*COL1A2* and -*AKT3* transgenic mice, the three proteins (PI3K, mTOR, and GSK3β) involved in the PI3K-AKT3 signaling pathway were significantly lower than that in the WT mice (*p* < 0.05), while MMP was significantly higher (*p* < 0.0001) (Figure 4B).

**SCHEME 1:**
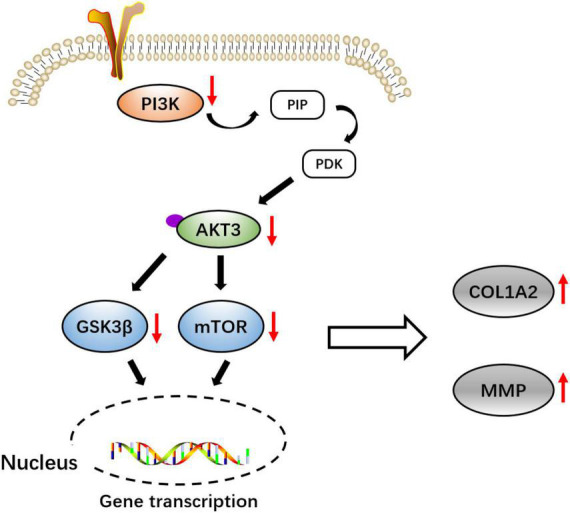
Schematic diagram of the molecular pathway of *COL1A2* and *AKT3* might be involved in the pathogenicity of congenital talipes equinovarus (CTEV).

Furthermore, we measured the relative expression of *PI3K*, *mTOR*, *GSK3*β, and *MMP* genes at the transcriptional level. In line with the Western blot results, in both the +*COL1A2* and -*AKT3* transgenic mice, the relative expression of *PI3K*, *mTOR*, and *GSK3*β genes involved in the PI3K-AKT3 signaling pathway was significantly reduced compared with the WT mice (*p* < 0.05) (Figures 4C–E), and MMP was significantly increased (*p* < 0.0001) (Figure 4F). Therefore, the PI3K-AKT3 signaling pathway was important and disrupted in the CTEV, which was in agreement with the KEGG analysis (Scheme 1).

## Discussion

mRNA sequencing was used to identify the key genes involved in the CTEV. After the sequencing and analysis, 10 most significantly upregulated and downregulated genes were selected. Of note, similar to *COL1A1* and *COL9A1*, the *COL1A2* gene is a member of the collagen family genes that play a vital role in osteogenesis, bone toughness, and bone metabolism (19). *COL1A1* and *COL1A2* encode the pro-α1 and pro-α2 chains of collagen type I, respectively, the major component of bone extracellular matrix (13). It has been reported that the abnormal expression or mutation of *COL1A2* may involve in the development of osteopenia and osteoporosis (20). Other upregulated genes such as *SPP1* encodes the protein of osteopontin, one of the non-collagenous, extracellular agglutinating glycoprotein located inside the bone, which may play a key role in the osteoclast attachment to the bone during bone resorption (21). In addition, osteopontin can regulate the proliferation, motility, migration, and blood vessel formation of human umbilical vein endothelial cells and then may lead to the occurrence of CTEV (22). It was interesting that the processes of hydrogen peroxide metabolic and oxygen transport were upregulated from the GO analysis results (Table 2). In line with the data from another study, Altay et al. measured the oxidative status of 38 patients with CTEV and found that the high levels of serum prolidase activity, total oxidant status, and oxidative stress index might be closely associated with the development of CTEV (23). From the KEGG pathway analysis results, we found that some genes involved in malaria, cancer, or other disease were upregulated in the patients with CTEV compared with the control children (Table 3). Malaria remains a highly prevalent disease that is caused by *Plasmodium falciparum* infection, which is often associated with procoagulant tonus characterized by thrombocytopenia and activation of the coagulation cascade and fibrinolytic system (24). Thus, malaria may induce angiogenesis, resulting in vascular malformation. However, lots of studies have proved that vascular abnormalities might play key roles in CTEV (2). Caspases are part of a family of cysteine proteases that are closely related to apoptosis, necrosis, and inflammation processes, which usually lead to cancers. In 2005, Heck et al. first found that caspase activity seemed to be related to the development of limbs, and some related genes were associated with CTEV (25). Therefore, the related pathways involved in malaria, cancer, and several other diseases may also associate with CTEV.

In addition, emerging evidence indicate that the PI3K-AKT3 signaling pathway plays a key role in cell proliferation, growth, survival, and metabolism (26). Of note, the PI3K-AKT3 signaling pathway involves the regulation of extracellular matrix and chondrocyte changes by inhibiting the MMP and protecting against the degradation of extracellular matrix (27). MMP was regulated by the PI3K-AKT3 signaling pathway and was one of the most important proteases that degrade extracellular matrix proteins including collagen and elastin and also related to other severe diseases, such as cancer (28–30). For other downregulated genes, *RPS27* is a ribosomal protein-related gene that can regulate the activity of p53 by binding with the murine double minute 2 (MDM2), and the p53-MDM2 pathway is an important regulatory mechanism leading to cell cycle arrest or apoptosis (31). Moreover, downregulation of *RPS27* may activate caspase 3 and then affect apoptosis by regulating the transcription of growth and apoptosis-related genes in the CTEV (32). In addition, there are also several limitations of this study. First, we screened at least 10 genes that were remarkably upregulated or downregulated between the CTEV and control samples according to the mRNA sequencing. But we have not probed the involved relationships among other genes with CTEV except the *COL1A2* and *AKT3* genes. In addition, the present data provide potential genes related to CTEV, which are not complete and adequate to reveal the pathogenesis of CTEV. More studies should be employed to investigate the in-depth reasons and mechanisms of CTEV.

In summary, we investigated the DEGs in child patients with CTEV compared with normal children using mRNA sequencing, and the gene of *COL1A2* was upregulated, and *AKT3* was downregulated at the transcriptional level. Western blot and qRT-PCR results also showed that the expression of *COL1A2* in CTEV was enhanced, and the *AKT3* was decreased. Moreover, the + *COL1A2* and -*AKT3* transgenic mice were constructed to prove that these two genes were truly closely related to the CTEV. In line with the KEGG analysis data, the PI3K-AKT3 signaling pathway might play a potentially important role in the regulation of pathological changes of CTEV.

## Data availability statement

The original contributions presented in this study are included in the article/Supplementary Material, further inquiries can be directed to the corresponding author/s.

## Ethics statement

The studies involving human participants were reviewed and approved by the Medical Ethics Committee of Shengjing Hospital affiliated to China Medical University (No. 2017PS226K). The patients/participants provided their written informed consent to participate in this study. The animal study was reviewed and approved by the Medical Ethics Committee for animal experiments of Shengjing Hospital affiliated to China Medical University.

## Author contributions

NW, JZ, and ZL contributed to the conception and design of the study. NW organized the database and wrote the first draft of the manuscript. HL performed the statistical analysis. JZ, HL, and ZL wrote sections of the manuscript. All authors contributed to manuscript revision, read, and approved the submitted version.

## Conflict of interest

The authors declare that the research was conducted in the absence of any commercial or financial relationships that could be construed as a potential conflict of interest.

## Publisher’s note

All claims expressed in this article are solely those of the authors and do not necessarily represent those of their affiliated organizations, or those of the publisher, the editors and the reviewers. Any product that may be evaluated in this article, or claim that may be made by its manufacturer, is not guaranteed or endorsed by the publisher.
