# Supplementary figures and images for: Regulation of COL1A2, AKT3 genes, and related signaling pathway in the pathology of congenital talipes equinovarus
Source: Front Pediatr. 2022 Jul 22;10:890109. doi: 10.3389/fped.2022.890109 (PMC9355787; doi:10.3389/fped.2022.890109)

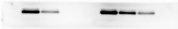

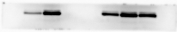

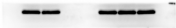

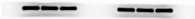

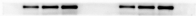

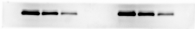

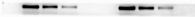

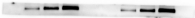

Supplement: Supplementary file 1 [file Data_Sheet_1.PDF]
